# Retraction: Iron-selenide-based titanium dioxide nanocomposites as a novel electrode material for asymmetric supercapacitors operating at 2.3 V

**DOI:** 10.1039/d6na90072k

**Published:** 2026-09-21

**Authors:** Muhammad Zia Ullah Shah, Hongying Hou, Muhammad Sajjad, Muhammad Sanaullah Shah, Kashif Safeen, A. Shah

**Affiliations:** a Faculty of Materials Science and Engineering, Kunming University of Science and Technology Kunming 650093 China hongyinghou@kust.edu.cn; b National Institute of Lasers and Optronics College, Pakistan Institute of Engineering and Applied Sciences Nilore Islamabad 45650 Pakistan attashah168@gmail.com; c College of Chemistry and Life Sciences, Zhejiang Normal University Jinhua 321004 P. R. China sajjadfisica@gmail.com; d Department of Physics, Abdul Wali Khan University Mardan 23200 KPK Pakistan

## Abstract

Retraction of ‘Iron-selenide-based titanium dioxide nanocomposites as a novel electrode material for asymmetric supercapacitors operating at 2.3 V’ by Muhammad Zia Ullah Shah *et al.*, *Nanoscale Adv.*, 2023, **5**, 1465–1477, https://doi.org/10.1039/D2NA00842D.

The Royal Society of Chemistry hereby wholly retracts this *Nanoscale Advances* article due to concerns with the reliability of the data.

The XRD patterns and Raman data in Fig. 1, and the EDX data in Fig. 3 were first published in ref. 1 by different authors. The SEM data in Fig. 2b is partially identical to data published in ref. 1 and has signs of being edited.

The authors provided a response, but it did not address the concerns. The authors were unable to provide the raw data.

Given the significance of these concerns, the findings presented in this paper are no longer reliable.

The authors did not respond to any correspondence regarding the retraction of this article.

Signed: Jeremy Allen, Executive Editor, *Nanoscale Advances*

Date: 9th September 2026
